# Mucosal transmissibility, disease induction and coreceptor switching of R5 SHIV_SF162P3N_ molecular clones in rhesus macaques

**DOI:** 10.1186/1742-4690-10-9

**Published:** 2013-01-31

**Authors:** Wuze Ren, Alexandra Mumbauer, Ke Zhuang, Carole Harbison, Heather Knight, Susan Westmoreland, Agegnehu Gettie, James Blanchard, Cecilia Cheng-Mayer

**Affiliations:** 1 Aaron Diamond AIDS Research Center, 455 First Ave., 7th Floor, New York, NY 10016, USA; 2 Division of Comparative Pathology, New England Primate Research Center, Harvard Medical School, Southborough, MA 01772, USA; 3 Tulane National Primate Research Center, Tulane University Medical Center, 18702 Three Rivers Road, Covington, LA 70433, USA

**Keywords:** R5 SHIV molecular clone, Coreceptor switch, Antiviral antibody response, Macrophage infection

## Abstract

**Background:**

Mucosally transmissible and pathogenic CCR5 (R5)-tropic simian-human immunodeficiency virus (SHIV) molecular clones are useful reagents to identity neutralization escape in HIV-1 vaccine experiments and to study the envelope evolutionary process and mechanistic basis for coreceptor switch during the course of natural infection.

**Results:**

We observed progression to AIDS in rhesus macaques infected intrarectally with molecular clones of the pathogenic R5 SHIV_SF162P3N_ isolate. Expansion to CXCR4 usage was documented in one diseased macaque that mounted a neutralizing antibody response and in another that failed to do so, with the latter displaying a rapid progressor phenotype. V3 loop envelop glycoprotein gp120 sequence changes that are predictive of a CXCR4 (X4)-using phenotype in HIV-1 subtype B primary isolates, specifically basic amino acid substations at positions 11 (S11R), 24 (G24R) and 25 (D25K) of the loop were detected in the two infected macaques. Functional assays showed that envelopes with V3 S11R or D25K mutation were dual-tropic, infecting CD4+ target cells that expressed either the CCR5 or CXCR4 coreceptor. And, consistent with findings of coreceptor switching in macaques infected with the pathogenic isolate, CXCR4-using variant was first detected in the lymph node of the chronically infected rhesus monkey several weeks prior to its presence in peripheral blood. Moreover, X4 emergence in this macaque coincided with persistent peripheral CD4+ T cell loss and a decline in neutralizing antibody titer that are suggestive of immune deterioration, with macrophages as the major virus-producing cells at the end-stage of disease.

**Conclusions:**

The data showed that molecular clones derived from the R5 SHIV_SF162P3N_ isolate are mucosally transmissible and induced disease in a manner similar to that observed in HIV-1 infected individuals, providing a relevant and useful animal infection model for in-depth analyses of host selection pressures and the *env* evolutionary changes that influence disease outcome, coreceptor switching and vaccine escape.

## Background

The human immunodeficiency virus (HIV) enters target cells through binding of its envelope glycoprotein gp120 to the human CD4 receptor and a coreceptor, either CXCR4 (X4 HIV) or CCR5 (R5 HIV) [1]. Over 80% of HIV-1 transmissions are initiated with R5 viruses [2-6], with X4 or dual-tropic viruses that use both CCR5 and CXCR4 (R5X4) emerging and coexisting with R5 viruses in 40-50% of non-treated subtype B and D infected individuals late in infection, but less often in subtype A and C infected patients [2,7-11]. Emergence of CXCR4-using viruses is frequently accompanied by rapid peripheral CD4+ T cell loss and progression to end-stage disease [12], but the mechanism(s) underlying their expansion is not well understood. It has been suggested that X4 viruses evolve from transmitted R5 viruses to broaden target cell populations [13], transitioning via an intermediate stage with respect to the envelope sequence and phenotypic characteristics [14,15]. However, early X4 presence has been documented in HIV-1 infection both phenotypically and genotypically [11,16-23], the latest from analysis of transmission clusters [24], with suppression of their replication following the development of HIV-specific immunity [16-18]. The finding that recently emerged CXCR4-using variants in some HIV-1 infected individuals are more neutralization sensitive than coexisting R5 viruses further supports a role of immune-mediated selection pressure against X4 virus [25]. Transmission and selection against CXCR4-using SIVsm in vivo [26], as well as emergence in the chronic phase of infection of variants that were transmitted but were not maintained at detectable levels early in infection have also been reported in SIV [27] and SHIV [28] infected monkeys. Thus, the possibility exists that R5-to-X4 conversion late in infection is the result of re-emergence of co-transmitted X4 or R5X4 variants when the immune system collapses.

Experimental infection of Asian macaques with simian (SIV) or simian-human immunodeficiency (SHIV) viruses are recognized as playing a critical role in advancing our understanding of HIV-1 transmission, pathogenesis, as well as basic vaccine, prevention and treatment concepts [29-31]. SHIVs that express the HIV-1 envelope glycoproteins (Envs) are particularly suited as challenge viruses to evaluate the role of viral tropism in AIDS pathogenesis and neutralizing antibody protection in the macaque model. The early pathogenic SHIVs primarily expressed CXCR4-using Envs and induced a disease course that differed from those observed in HIV-1 infected individuals and SIV-infected rhesus [32-35]. Subsequently, several clade B and C R5 SHIVs have been constructed that showed varying degree of mucosal transmissibility, replication competence and pathogenicity [34,36-40], but R5 SHIV molecular clones that induce a HIV-1 like pathology including coreceptor switch coincident with peripheral CD4 decline and predominance of macrophage tropism at end-stage disease have not been described. We developed a model of experimental infection of Asian macaques with the late-stage SHIV_SF162P3N_ isolate that exhibited many similarities to HIV-1 infection in humans including CCR5 coreceptor usage, mucosal transmissibility, acute depletion of mucosal memory CD4+ T cells, persistent infection, and progression to AIDS over a period of several months to years in a proportion of the infected animals [41,42]. Moreover, expansion or switch to CXCR4 was observed in ~50% of R5 SHIV_SF162P3N_-infected AIDS macaques, with viruses that can function with both coreceptors serving as intermediates [43,44]. Similar to HIV-1, the main determinants for coreceptor usage of SHIV_SF162P3N_ are located in the third (V3) variable loop of Env [43,44]. However, whereas coreceptor switch in most HIV-1 infected individuals occurred following the development of antiviral antibodies, the macaques in which coreceptor switch was observed were primarily rapid progressors (RPs) that failed to mount or sustain an antiviral antibody response. Thus, there is concern that the selective pressures for phenotypic conversion in the RP macaques might not fully reflect the human situation. Furthermore, because the populations in the SHIV_SF162P3N_ virus stock are comprised of related but not identical variants, the presence of low-level X4 viruses in the inoculum that were initially co-transmitted and remaining cryptic until immune selective pressure is sufficiently diminished cannot be excluded. For these reasons, we generated molecular clones of R5 SHIV_SF162P3N_ for intrarectal inoculation, with the objective of documenting disease development and a shift in coreceptor preference during the course of natural infection. Mucosally transmissible, highly replication competent and pathogenic R5 SHIV molecular clones would also be very useful as challenge viruses in vaccine efficacy and escape studies.

## Results

### Envelope sequence and function of SHIV_SF162P3N_ molecular clones

**Figure 1:**
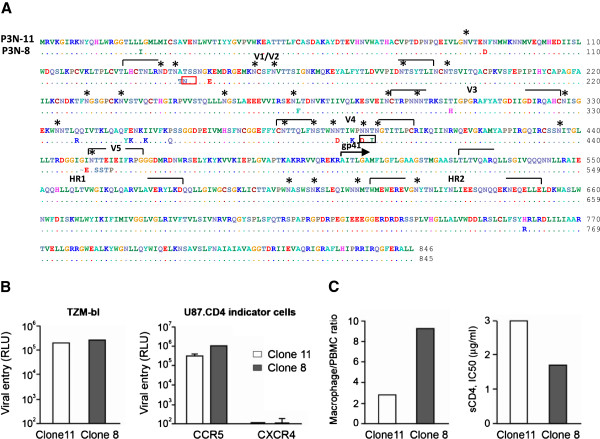
**Envelope sequence and function of R5 SHIV**_**SF162P3N **_**molecular clones. (A)** Comparison of envelope gp160 sequence of SHIV_SF162P3N_ clones 8 and 11. Dots denote identical residues in the sequence and * indicates potential N-linked glycosylation sites (PNGSs). PNGSs that are absent or re-positioned in clone 8 envelope gp120 are designated by black and red boxes, respectively. **(B)** Entry into TZM-bl cells and U87.CD4 indicator cell lines, and **(C)** sCD4 sensitivity and infection of primary macrophages that express low levels of CD4 with pseudotyped viruses bearing clone 8 and 11 Env gp160. Infectivity in macrophages was expressed as a ratio of infectivity in autologous PBMCs that express high levels of CD4 and CCR5. RLU, relative light unit. All viral entry and infectivity experiments were tested in triplicates. Data shown are the means and standard deviations from triplicate wells and are representative of at least two independent experiments.

The V3 loop of envelope gp120 plays a major role in coreceptor usage, tropism and neutralization susceptibility, factors that modulate viral pathogenesis [45]. Accordingly, two major V3 loop species, each representing 14 of 43 envelope clones sequenced in the R5 SHIV_SF162P3N_ virus quasispecies [41], were chosen for construction of SHIV_SF162P3N_ molecular clones to assess their utility in studies of lentiviral pathogenesis and AIDS vaccine. Comparison of the *env* gp160 of the two molecular clones showed differences only in gp120. The net positive charge for the V3 variable loop of clone 8 is +5 as compared to +4 for clone 11, with notable differences between the two clones in the V4 and V5 domains, and in the potential N-linked glycosylation sites (PNGSs) as well. Specifically, there was a repositioning of a PNGS in the V1V2, with a loss of PNGS in the V4 domain of clone 8 gp120 as compared to clone 11 gp120 (Figure 1A). Both Envs function only with CCR5, infecting U87.CD4 cells expressing CCR5 but not CXCR4, with no significant difference in their entry efficiency into TZM-bl cells that express high levels of CD4 and CCR5 (Figure 1B). However, clone 8 infected primary macrophages more efficiently, and was 2-fold more sensitive to neutralization with sCD4 than clone 11 (90% inhibitory concentration IC_90_ 1.7 μg/ml vs 3.0 μg/ml; Figure 1C), suggesting that it binds to the CD4 receptor with higher affinity.

### R5 SHIV_SF162P3N_ molecular clones are infectious by the intrarectal route and induce disease

**Figure 2:**
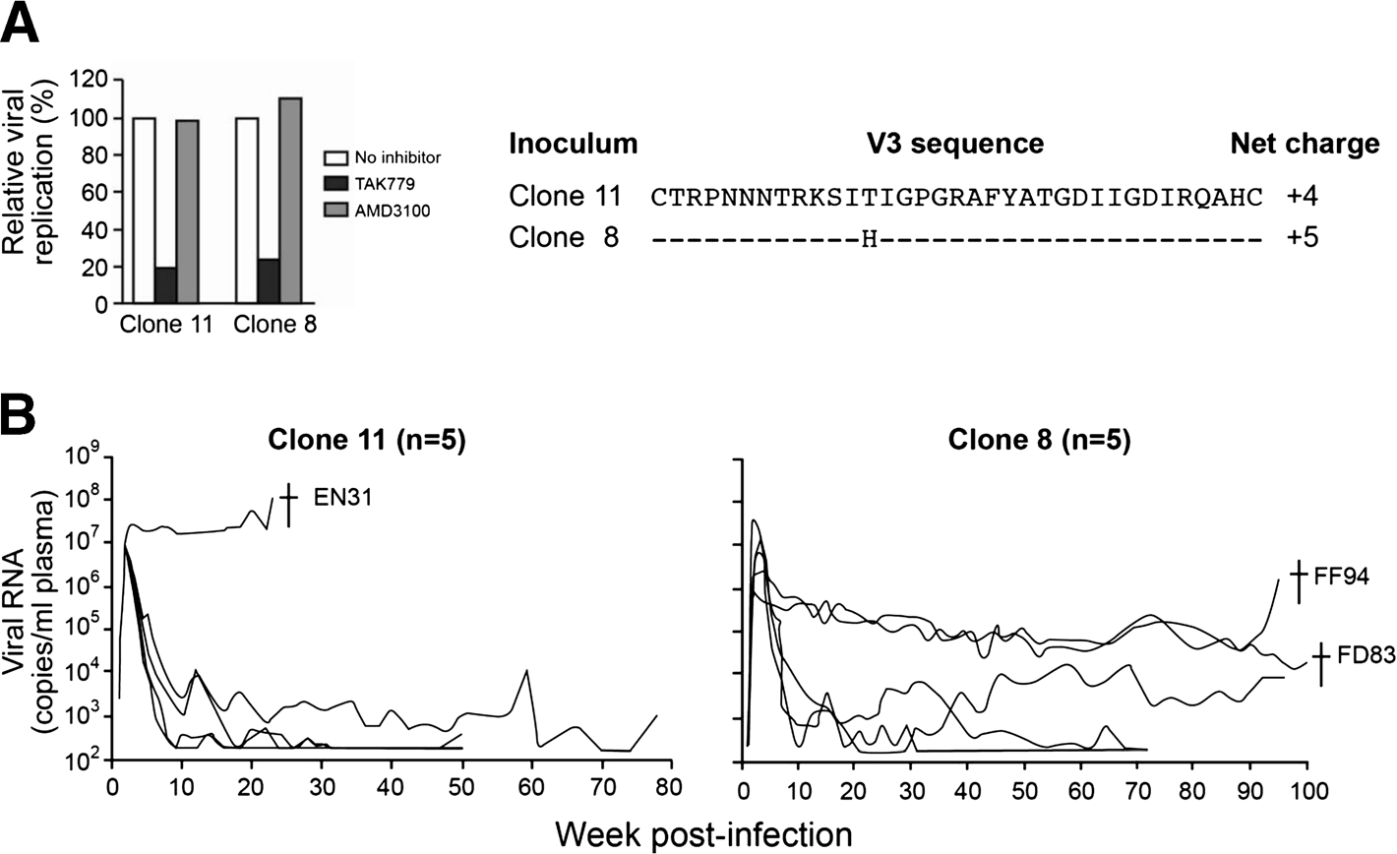
**SHIV**_**SF162P3N **_**clone 8 and 11 replication in intrarectally challenge macaques. (A)** Coreceptor usage in rhPBMC and the V3 loop sequence of SHIV_SF162P3N_ clone 8 and 11, and **(B)** plasma viral RNA levels over time in the infected animals are shown. Coreceptor usage of the inoculating viruses was determined by blocking entry into rhPBMC with 1 μM CXCR4-specific (AMD3100) or CCR5-specific (TAK779) inhibitor. For V3 loop sequence, dashes stand for identity in sequences, and the net positive charge of this region is shown on the right. † in (B) indicates euthanasia with clinical symptoms of AIDS.

We next tested the mucosal transmissibility and pathogenicity of SHIV_SF162P3N_ clones 8 and 11. We confirmed CCR5 usage of the two molecular clones in rhPBMCs by demonstrating that the CCR5 inhibitor TAK779 and not the CXCR4 inhibitor AMD3100 blocked replication of these viruses (Figure 2A). Five of five macaques inoculated intrarectally with clone 8 or 11 were productively infected, with peak viremia of 6–7 log_10_ RNA copies/ml plasma (Figure 2B). Four of the five clone 11-infected macaques controlled their infection to levels ≤ 3 log_10_ RNA copies/ml plasma after 20 weeks of infection, with one, EN31, sustaining high viral load (>7 log_10_ RNA copies/ml plasma). EN31 developed clinical symptoms of AIDS, and was euthanized at 23 weeks post-infection (wpi). In comparison, while virus replication also declined in the post-acute phase in three of the five clone 8-infected macaques, a rebound to levels of 4 log_10_ RNA copies/ml plasma was seen in one of these three animals at 40 wpi. Moreover, the remaining two clone 8-infected monkeys maintained a steady state level of 5 log_10_ RNA copies/ml plasma, with development of disease at 95 and 100 wpi (FF94 and FD83, respectively). These results show that both R5 SHIV_SF162P3N_ molecular clones are mucosally transmissible and are pathogenic, but viremia appeared to be more persistent in the clone 8- than the clone 11-infected macaques.

### Severe lymph node CD4 T cell loss in macaques infected with R5 SHIV_SF162P3N_ molecular clones that developed disease

**Figure 3:**
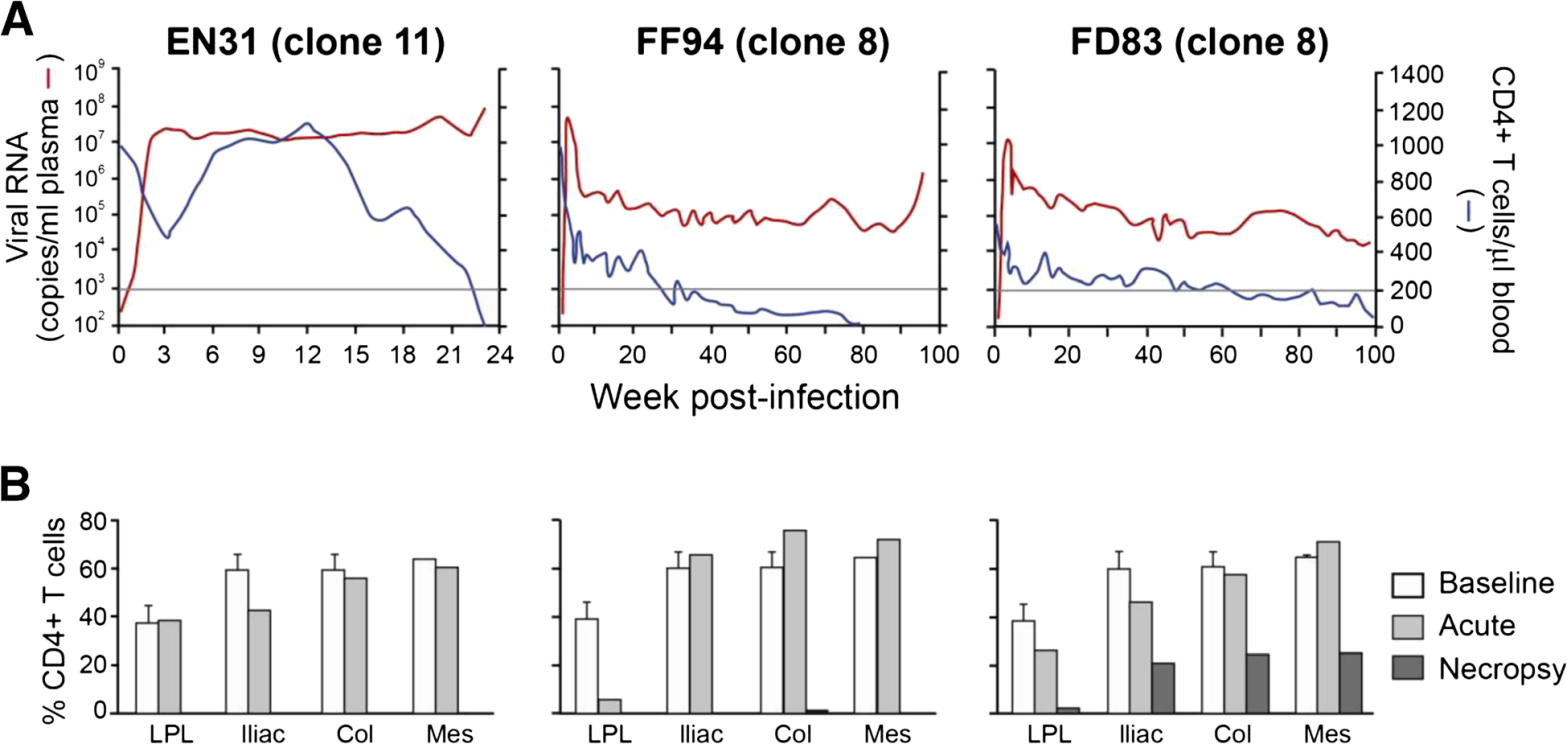
**Severe CD4+ T cell loss in RMs infected with SHIV**_**SF162P3N **_**molecular clones. (A)** Plasma viral load and peripheral CD4+ T cell count in RMs that developed clinical symptoms of AIDS. Horizontal line denotes CD4+ T cell count of 200 cells/ul blood. **(B)** Percentages of CD4+ T cells in the lamina propria lymphocyte (LPL) from the jejunum, iliac, colonic (col) and mesenteric (Mes) lymph nodes during acute viremia (2 wpi) and at time of necropsy. Baseline values generated from three uninfected macaques are shown for reference.

Analysis of peripheral CD3 + CD4+ T cells in the three macaques (EN31, FF94, FD83) that progressed to disease showed transient loss in the clone 11-infected macaque EN31 during peak viremia (2–3 wpi) that rebounded to baseline levels at 6 wpi (Figure 3). By 12 wpi, however, peripheral CD4+ T cell count began to decline, with precipitous drop to < 2 cells/μl blood at end-stage disease. Peripheral CD4+ T cell loss was more gradual and protracted in the two clone 8-infected animals. At the time of euthanasia, CD4+ T cell count in FF94 and FD83 was 5 and 61 cells/μl blood, respectively. Significant acute depletion (>70% loss) in tissue CD4+ T lymphocytes was observed only in the lamina propria (LP) of the intestine of FF94, with minimal loss in the peripheral lymph nodes (LNs) of all three animals (Figure 3B). The more dramatic acute depletion of gut CD4 cells in FF94 as compared to EN31 and FD83 may be related to the fact that surgery was performed one week later in FF94 (3 wpi) than in the other two. At the time of necropsy, however, >99% of CD4+ T lymphocytes in the gut of all three macaques were depleted. Massive depletion of CD4+ T cells was also seen in the LNs of EN31 and FF94 at the time of euthanasia, but 20-35% of this T cell subset was preserved in secondary lymphoid tissues of FD83. The severe loss in peripheral as well as lymphoid CD4+ T lymphocytes in EN31 and FF94 at the time of euthanasia is suggestive of X4 presence, prompting us to analyze envelope sequences in the tissues of these animals.

### Coreceptor switch in macaques infected with R5 SHIV_SF162P3N_ molecular clones

**Figure 4:**
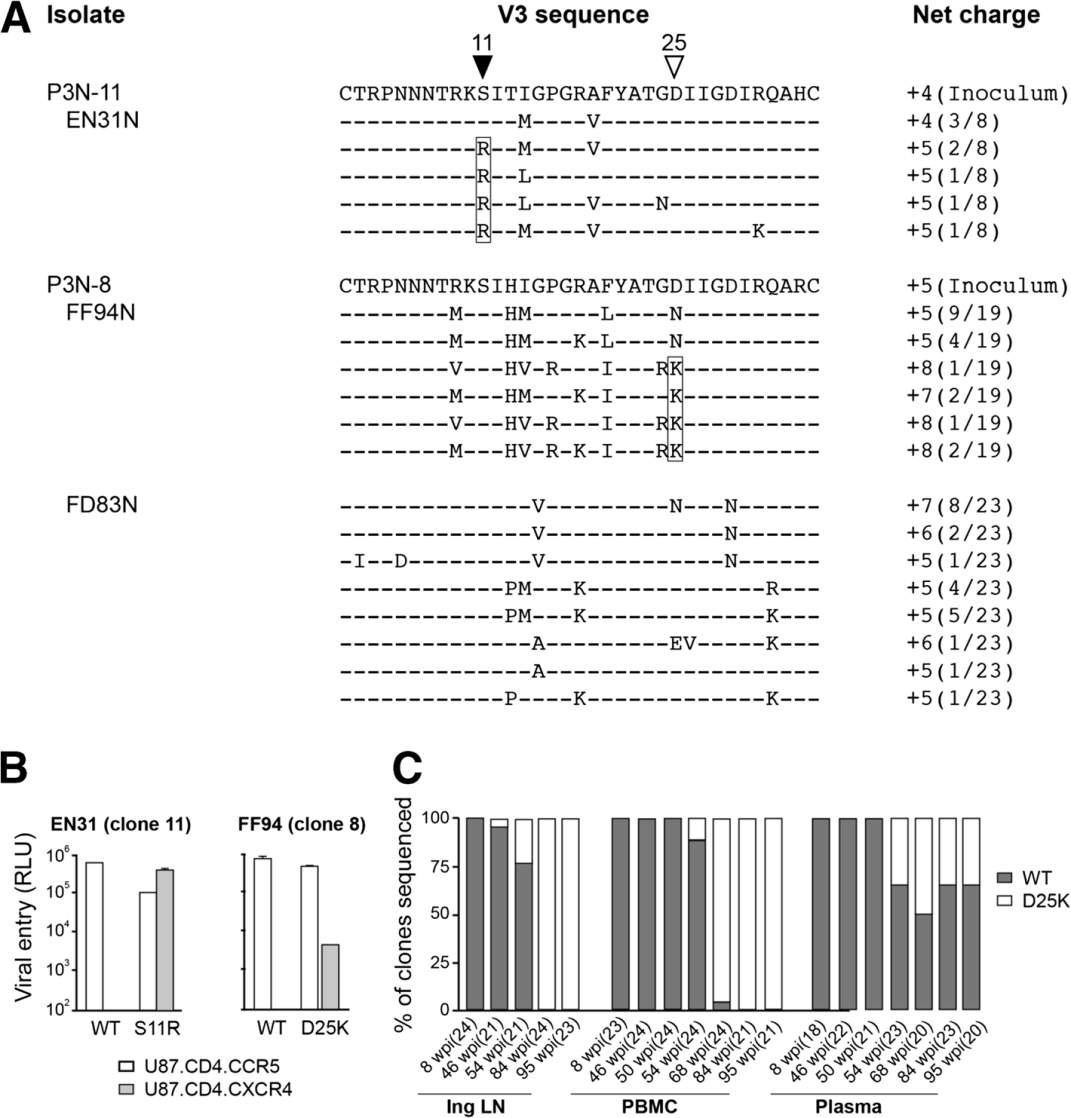
**V3 loop sequence (A), coreceptor usage (B) and distribution (C) of viruses in macaques infected with R5 SHIV**_**SF162P3N **_**molecular clones. (A)** V3 loop sequence comparison of the inoculating clonal virus and representative lymph node viruses in macaques EN31 (clone 11; colonic), FF94 (clone 8; mesenteric) and FD83 (clone 8; colonic) at time of necropsy. Dashes stand for identity in sequences, and the net positive charge of this region is shown on the right. Positions 11 and 25 within the V3 loop are indicated by triangles, with brackets highlighting the presence of positively charged amino acids at these positions. The numbers in parentheses represent the numbers of clones matching the indicated sequence per total number of clones sequenced. **(B)** Infection of U87.CD4.CCR5 and U87.CD4.CXCR4 indicator cells with pseudovirions bearing S11R, D25K or WT V3 sequences. RLU, relative light units. Data are the means and standard deviations from triplicate wells and are representative of at least two independent experiments. **(C)** Distribution of viruses in Inguinal LN, PBMC and plasma of FF94 over time as determined by clonal sequence analysis. Numbers in parentheses indicate number of gp120 clones sequenced at each time point.

Envelope sequence analysis revealed that, in contrast with FD83, five of eight clones amplified from the lymph node of EN31 at the time of necropsy harbored arginine at position 11 of the V3 loop (S11R), while those in FF94 had, at position 25, either lysine (D25K; 23 of 24 clones) or arginine (D25R; 1 of 24 clones) residue (Figure 4A). Basic amino acid substitutions at these positions of the V3 loop are strongly associated with a CXCR4-using phenotype in HIV-1 infected individuals [46-49]. Moreover, substitution at position 25 of the V3 loop of FF94 was frequently accompanied by the introduction of an arginine at position 24 that had been reported to improve the predictive value of X4 presence in humans [50]. Functional assays showed that in contrast with Envs bearing WT V3 sequence that use only CCR5 for entry, Envs harboring V3 S11R (EnvS11R) or D25K mutation (EnvD25K) were dual-tropic, infecting U87.CD4 cells that expressed either the CCR5 or CXCR4 coreceptor (Figure 4B). Interestingly, deep sequencing of gp120 V3 in the clone 11 inoculum (21,048 reads) revealed presence of the S11R mutation at very low frequency (0.07%), raising the possibility of X4 evolution during clone expansion in vitro. S11R substitutions as a result of a single nucleotide point mutation were also detected in the SHIV_SF162P3N_ clone 8 virus stock (0.11%; 37,360 reads). But the D25K mutation associated with phenotypic switch in FF94 was absent, supporting evolution to CXCR4-use in vivo.

Peripheral lymph nodes were found to be the preferred sites of X4 emergence and expansion in macaques infected with R5 SHIVs [42,51,52]. Because longitudinal samples (w8, 46, 54, 84) from the inguinal lymph node (Ing LN) of FF94 were available, we analyzed this tissue compartment as well as the PBMC and serum samples collected at the corresponding time points for X4 emergence in this animal. We found that the dual-tropic D25K V3 variant was detected by clonal analysis at low frequency (1 of 21 clones sequenced) in the Ing LN of FF94 sampled at 46 wpi. Representation of this variant in the Ing LN increased over time, with all 24 clones sequenced harboring this mutation at 84 wpi (Figure 4C). The D25K variant was not detectable in the blood by clonal sequence at 46 and 50 wpi, emerging in plasma and PBMCs sampled four weeks later. Representation of the V3 variant increased over time in blood cells, becoming the major Env species from 68 wpi until the time necropsy. In contrast, the D25K variant co-existed with R5 viruses in the plasma at all time points analyzed. Collectively, the results confirm and extend our findings with the isolate [42], demonstrating mutational pathways to CXCR4-usage that overlapped with those seen in HIV-1 infected individuals, with X4 evolution and emergence first in secondary lymph nodes.

### Tropism switch following the development of a neutralizing antibody response

**Figure 5:**
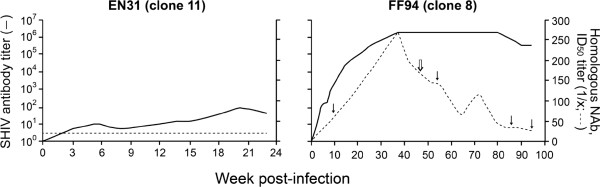
**SHIV-specific antibody response.** SHIV binding and homologous neutralizing antibody titers in EN31 (clone 11) and FF94 (clone 8) are shown. Closed arrows indicate time points when Ing LN biopsies were collected and analysed, with open arrow indicating time of X4 detection.

Both EN31 and FF94 seroconverted at 3 wpi. SHIV-binding antibody titers continued to increase in FF94, reaching peak endpoint titers >6 log_10_ at 30–40 wpi, but plateaued at 4–5 log lower levels in EN31 (Figure 5). EN31 also failed to mount a neutralizing antibody (NAb) response against the inoculating virus (clone 11), as has been described for some HIV-1 infected individuals [53]. Homologous NAb against the clone 8 virus however was detected in FF94 beginning at 12 wpi, and continued to increase, reaching 50% inhibitory dilution (ID_50_) titers >200 at 30–40 wpi. Titers declined thereafter, to ID_50_ levels <50 at the time of euthanasia. The high viral load, weak antiviral antibody response and disease development in EN31 within 30 weeks of infection classified this animal as a rapid progressor, while FF94 displayed a more typical disease course. These results with R5 SHIV_SF162P3N_ molecular clones show that similar to HIV-1 infected individuals, coreceptor switch in rhesus macaques occurs in rapid progressors as well as in conventionally infected animals that mounted a neutralizing antibody response. Moreover, X4 emergence in FF94 coincides with persistent peripheral CD4+ T cell-depletion below 200 cells/ul blood (Figure 3) and a decline in neutralizing titers against the inoculating virus (Figure 5) that are suggestive of immune deterioration.

### Tissue macrophages sustain virus production in FF94

**Figure 6:**
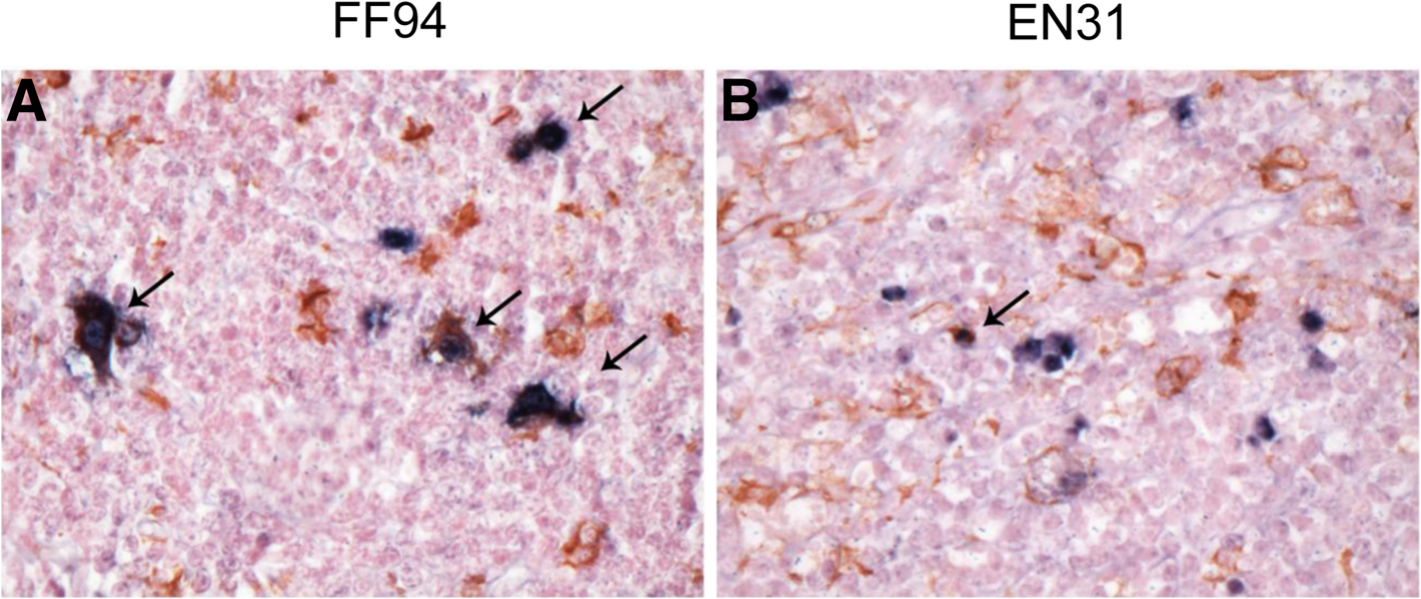
**Tissue macrophages are the primary SHIV-infected cells at end stage disease in FF94.** Double-labeled immunohistochemical staining of the mesenteric LN of FF94 and EN31 for SIVnef (brown) and the macrophage marker lba-1 (red) was performed. Arrows mark representative double-positive cells.

The persistence of high viral load, with a rise towards end-stage disease despite near complete depletion of peripheral CD4+ T cells in FF94 prompted us to investigate the source of virus production. Combined in situ hybridization and immunohistochemistry staining with SIVnef and Iba-1 showed that the majority of SHIV-infected cells in the mesenteric LN of FF94 (>85%) are lba-1 positive, indicating that they are of the macrophage lineage (Figure 6). In comparison, only ~35% of SIVnef + cells in EN31 are stained with the lba-1 antibody. Macrophages, therefore, are the principal virus-producing cells in FF94 at end-stage disease.

## Discussion

The origin of CXCR4-using variants has been investigated extensively. This is because X4 presence is known to be associated with poorer clinical prognosis, and is a major concern in the clinical use of CCR5 inhibitors [54-58]. Several lines of investigation support the hypothesis that X4 and R5X4 viruses evolved from pre-existing R5 variants which are the founder viruses in most cases of HIV-1 transmission. However, the bulk of the evidence in support of early R5 dominance in humans is made using blood from subjects who manifested clinical signs of acute infection syndrome, several weeks after the initial transmission event. Thus, the possibility that coreceptor switch during HIV-1 infection is the result of re-emergence of co-transmitted X4 or R5X4 viruses that reside in tissue sites that are not sampled or exist at levels below the threshold of detection cannot be excluded. Using a relevant nonhuman primate model of HIV-1 pathogenesis, we demonstrate in this study that R5 SHIV_SF162P3N_ molecular clones are efficiently transmitted to macaques via intrarectal inoculation, with disease induction and switch in coreceptor tropism following the development of a neutralizing antibody response. Furthermore, we obtain evidence suggestive of a role of antibody selective pressure in counteracting X4 evolution and expansion.

Molecular clones of subtype B [59] and subtype C [60,61] R5 SHIVs that are mucosally transmissible, highly replication competent and capable of inducing AIDS in rhesus macaques have been described, but expansion or conversion to CXCR4 usage has not been observed. In this regard, we show that both R5 SHIV_SF162P3N_ molecular clones exhibited coreceptor switching that followed the 11/25 rule derived from subtype B HIV-1 [47]. In the RP macaque EN31, serine at position 11 of the V3 loop was substituted with arginine, while in FF94, the chronic progressor that mounted an autologous neutralizing antibody response, the aspartic residue at position 25 was replaced with either lysine or arginine residues (Figure 4A). In 17/24 clones amplified from LN of FF94 at end-stage, this substitution at position 25 was accompanied by introduction of a charged residue at position 24 of the V3 loop that had been reported to improve the predictive value of X4 presence in humans [50]. Moreover, the D25K V3 mutational event in FF94 occurred first in the lymph node where naïve T cells that express high levels of CXCR4 are enriched, in agreement with data published by us and others that peripheral LNs are the prefer sites of X4 evolution and amplification [42,51,52]. Notably, emergence of the dual-tropic D25K variant in the LN was in the presence of neutralizing antibody (ID_50_ titers of ~150 against the inoculating clone 8 virus at 46 wpi; Figure 5), with clonal analysis of the viral quasispecies showing increasing dominance of the D25K V3 variant in LN and blood cells, but not in the plasma (Figure 4C). These findings are consistent with observations in HIV-1 infected individuals of a higher prevalence of X4 viruses in PBMCs compared to serum [62-64], and support the notion that X4 viruses emerge and predominate in body compartments with lower antibody pressure than in the plasma, spreading via cell-to-cell transmission that is less susceptible to antibody neutralization. Recently emerged CXCR4-using variants in some HIV-1 infected patients have been reported to be more neutralization sensitive than coexisting R5 viruses [25], implying that systemic dissemination is possible only with immune system erosion that decreases the selection pressure. X4 detection in peripheral blood of FF94 at 54 wpi coincides with persistent CD4+ T cell loss to levels <200 cells/μl blood (Figure 3A) and a decline in neutralizing titers against the homologous inoculating virus that are suggestive of immune erosion (Figure 5). It will be of interest to investigate neutralization sensitivity of the emerging and late CXCR4-using variants in FF94 to contemporary sera to link a decline in autologous neutralization antibody response with X4 emergence.

The infectious molecular clones were obtained by transfection of 293T cells but cultured and propagated in activated rhesus PBMC to generate virus stocks. This expansion process in vitro necessarily introduces mutation and diversity, raising the possibility that the V3 mutations identified with tropism switch in the infected animals might have already been present in the inoculum. Deep sequencing of V3 region in the SHIV_SF162P3N_ clone 11 virus stock indicated that this is indeed the case. Fifteen of over 21,000 sequences analyzed (0.07%) harbored the S11R mutation, raising the possibility that the R5X4 variant could have been co-transmitted and eventually outgrew in macaque EN31 which failed to mount a neutralizing antibody response. In contrast, while the S11R variant was also present in the SHIV_SF162P3N_ clone 8 virus stock (0.11%), it was not detected by clonal sequence analysis in the conventional macaque FF94 at terminal disease stage. It is conceivable that the S11R variant was not co-transmitted in this animal. Alternatively, it was co-transmitted but remained cryptic. Further studies are needed to examine these possibilities. But importantly, deep sequencing failed to reveal the presence of D25K mutation associated with tropism switch in FF94 in the clone 8 virus inoculum, consistent with findings that multiple long-term cultures of clonal virus variants on PBMCs results in only very few mutations in the V3-V4 regions [65]. We conclude, therefore, that the V3 mutations that confer CXCR4 usage in FF94 evolved from pre-existing R5 variants. Analysis of recombinant V3 mutant viruses showed that while the S11R mutation conferred efficient CXCR4 usage, the D25K V3 mutant entered CXCR4-expressing cells less efficiently than cells expressing CCR5, in agreement with findings in infected individuals that changes in V3 position 25 alone are not highly predictive of coreceptor switching. Besides the V3 loop, mutations in V1V2 domain of gp120 can also influence coreceptor choice [66-73], consistent with structural studies in which both V3 and the stem of the V1V2 loop were shown to participate in coreceptor binding [74-76]. Furthermore, in vitro studies suggested that the order of occurrence of mutations associated with coreceptor switching is critical for survival of the intermediates, with mutations in V1/V2 preceding those of V3 to permit virus survival [77]. Detailed analysis of *env* sequence changes over time in FF94, in particular the V1/V2 domain of gp120, and the relationship of these changes to autologous neutralizing antibody response and viral fitness should provide important insights into the requirements and constraints for evolution from CCR5 to CXCR4 use in vivo.

We previously reported sustained viremia and progression to disease over a one-year infection period in ten of eleven rhesus macaques infected intrarectally with high dose of the R5 SHIV_SF162P3N_ isolate, with a RP phenotype and coreceptor switching observed in four and five of the eleven monkeys with AIDS respectively [78]. In this regard, although the results with the molecular clones confirm and extend findings with the isolate, showing similar evolutionary pathways, dynamics and sites of X4 emergence [42-44], infection with the molecular clones is less pathogenic. The decrease in replicative capacity and pathogenicity of R5 SHIV_SF162P3N_ molecular clones may be related to the fact that they are less diverse than the isolate. Indeed, transmission of multiple viral variants has been suggested to influence viral persistence and rates of disease development through recombination to generate intrahost phenotypic and pathogenic diversities to escape early host selective pressures and increase fitness [79-82]. And, although the number of animals used is small, the findings suggest that SHIV_SF162P3N_ clone 8 may be more pathogenic than clone 11. A higher proportion of the clone 8-infected macaques sustained viremia (3 of 5) and progressed to disease (2 of 5) over a two-year observation period as compared to only one of five of the clone 11-infected monkeys. The envelope glycoproteins of the two clones differed in sCD4 sensitivity and as well as infection of macrophages that are long-lived viral reservoirs [83]. Macrophages have been reported to be the principal reservoir and sustain high viral loads in rhesus monkeys after the depletion of CD4+ T cells by highly pathogenic X4 SHIVs [84], and following coreceptor switch in R5 SHIV_SF162P3N_-infected RPs [85]. Infected macrophages are relatively resistant to CD8+ T cell-mediated suppression [86,87] and macrophage internal HIV-1 is protected from neutralization antibodies [88]. Combined in situ hybridization and immunohistochemical analysis of LNs obtained at the time of necropsy indicated higher viral burden in the LNs of FF94 than EN31, with a majority (>80%) of infected cells in the former co-staining with lba-1, a macrophage marker. Thus, it is tempting to speculate that the difference in macrophage infection by the two molecular clones contributed to their differences in viral persistence. Studies in additional animals will be needed to address this. Regardless, the data support the use of both SHIV_SF162P3N_ molecular clones to assess the efficacy of vaccines in preventing HIV-1 acquisition or in reducing peak viral load and virus-induced depletion of gut CD4+ T cells, but clone 8 may be more useful than clone 11 in judging the effects of vaccines in dampening the intensity of virus infection.

## Conclusions

In summary, this report documents coreceptor switch in macaques infected with R5 SHIV molecular clones, supporting R5 evolution to X4. Coreceptor switch in macaques infected with R5 SHIV_SF162P3N_ molecular clones required genetic adaptations similar to those seen in humans, and occurred in rapid as well as conventional progressors that mounted a neutralizing antibody response. This animal model, in which the envelope sequence and functional properties of the inoculating virus are known, and where detailed samplings of blood and tissue samples are possible, provides the unique opportunity to uncover in detail the genetic requirement, obstacles and constraints for virus phenotype evolution in vivo. It can also be used to study neutralization escape during the course of infection and determine the role of humoral immunity in X4 virus emergence. Lastly, because the molecular clones express R5 HIV-1 envelope glycoproteins, main targets for neutralizing antibodies, they are better suited than the X4 SHIVs and SIVs that differ antigenically from HIV-1 as challenge viruses for antibody-based vaccine testing and development.

### Materials and methods

#### Cells

293T cells, TZM-bl cells expressing CD4, CCR5 and CXCR4, and containing integrated reporter genes for firefly luciferase and β-galactosidase under control of the HIV-1 LTR [89] were propagated in DMEM supplemented with 10% fetal bovine serum (FBS), penicillin, streptomycin and L-glutamine. U87 cells stably expressing CD4 and one of the chemokine receptors [90] were maintained in DMEM supplemented with 10% FBS, antibiotics, 1 μg/ml puromycin (Sigma-Aldrich, St. Louis, MO) and 300 μg/ml G418 (Geneticin; Invitrogen, Carlsbad, CA). Rhesus peripheral blood mononuclear cells (PBMCs) were prepared by Ficoll gradient centrifugation, stimulated with staphylococcal enterotoxin B (SEB, 3 μg/ml ; Sigma-Aldrich), and cultured in RPMI medium containing 10% FCS, penicillin, streptomycin, L-glutamine and 20U/ml interleukin-2 (Norvatis, Emeryville, CA). Monocytes were enriched by centrifugation of human PBMCs through a 40% percoll cushion followed by plastic adherence, and cultured in RPMI 1640 medium supplemented with 10% FCS and 5% human AB serum for 5–7 days to allow for differentiation into macrophages [91].

### Construction of R5 SHIV_SF162P3N_ molecular clones

Full-length gp160 coding sequences of the R5 SHIV_SF162P3N_ inoculum were obtained and confirmed as previously described [41], and subcloned into the corresponding region of the 3’SHIV_SF162_ genome [34] using the unique *Kpn* I and *Xho* I sites. Infectious molecular clones were recovered by cotransfection of 293T cells with a ligation product of the 3’ SHIV_SF162 P3N_ gp160 and the 5’ SIV hemigenomes, followed by cocultivation with SEB-stimulated rhesus PBMCs. Stocks of SHIV_SF162P3N_ molecular clones were propagated and tittered in rhesus PBMCs.

### Animal inoculation and clinical assessments

All inoculations were carried out in adult rhesus monkeys of Indian origin (*Macaca mulatta*) housed at the Tulane National Primate Research Center (TNRPC) in compliance with the *Guide for the Care and Use of Laboratory Animals*. Animals were confirmed to be serologically and virologically negative for simian type D retrovirus, and serologically negative for SIV and simian T-cell lymphotropic virus prior to infection, and were screened for the presence of the Mamu-A*01, Mamu-B*17 and Mamu-B*08 class I alleles previously shown to be associated with control of pathogenic SIVmac239 replication using standard PCR with allele-specific primers [92]. Macaques received a single intrarectal (ir) inoculation with 5 x 10^3^ 50% tissue culture infectious dose (TCID_50_) of the cell free challenge stocks of SHIV_SF162P3N_ molecular clones. Whole blood from the inoculated animals was collected weekly for the first eight weeks, biweekly for another 16 weeks, and monthly thereafter. Surgery was performed at peak (2–3 weeks post-infection, wpi) viremia for collection of tissues from one external and one internal lymph node, and from internal organs such as the small intestine, bone marrow, thymus and spleen. Animals were euthanized at end of study period by intramuscular administration of telazol and buprenorphine followed by an overdose of sodium pentobarbital, and tissues from multiple sites were collected. Euthanasia was considered to be AIDS related if the animal exhibited peripheral blood CD4+ T-cell depletion (<200/mm^3^), greater than 25% loss of body weight and combinations of the following conditions: diarrhea unresponsive to treatment, opportunistic infections, peripheral lymph node atrophy, and abnormal hematology (e.g., anemia, thrombocytopenia, or leukopenia). Plasma viremia was quantified by branched DNA analysis (Siemens Medical Solutions Diagnostic Clinical Lab, Emeryville, CA) and absolute CD4+ and CD8+ cell counts were monitored by TruCount (BDBiosciences, Palo Alto, CA). The percentages of CD4+ T cells in the tissue cells were analyzed by flow cytometry (FACScalibur) using CD3-fluorescein isothiocyanate (FITC), CD4-phycoerythrin (PE) and CD8- peridinin chlorophyll protein (PerCP) antibodies. Except for CD3-FITC (BioSource, Camarillo, CA), all antibodies were obtained from BD Biosciences.

### Envelope sequence analysis

For sequence analysis of V3 variants in PBMCs and tissues, proviral DNA was extracted from 3 x 10^6^ cells with a DNA extraction kit, and the V1 to V5 region of gp120 was amplified from the vDNA using Taq DNA polymerase (Qiagen, Chatsworth, CA) with primers ED5 and ED12 or ES7 and ES8 as previously described [93]. For sequence analysis of variants in the plasma, viral RNA was prepared from 300–500 μl plasma using a commercially available RNA extraction kit (Qiagen) followed by reverse-transcription (RT) with Superscript III RT (Invitrogen, Carlsbad, CA) and random hexamer primers (Amersham Pharmacia, Piscataway, NJ), with amplification of the V1 to V5 region of gp120 from the RT products. The PCR products were cloned with the TOPO TA cloning kit (Invitrogen) per the manufacturer’s instructions followed by direct automated sequencing (Genewiz, South Plainfield, NJ). The sequences obtained were aligned with Clustal X [94], edited manually using BioEdit V7.0.9 and translated to the amino acid sequence. Deep sequencing of the inoculating virus stocks was performed using the Solexa Illumina platform by the Genomic Resource Center at the Rockefeller University.

### Envelope expression plasmid construction and pseudotype virus production

The generation of envelope (Env) expression plasmids, V3 mutants and luciferase-reporter viruses have been described previously [42]. Briefly, full length gp160 coding sequence was amplified with primers SH43 (5’-AAGACAGAATTCATGAGAGTGAAGGGGATCAGGAAG-3’) and SH44 (5’-AGAGAGGGATCCTTATAGCAAAGCCCTTTCAAAGCCCT-3’), and subcloned into the pCAGGS vector. Site-directed mutagenesis was employed to introduce specific V3 mutations into the backbone of the parental Envs, and *trans*-complementation assay was then used to generate luciferase reporter viruses capable of only a single round of replication. The pseudovirions were quantified for p24gag content (Beckman Coulter, Fullerton, CA).

### Determination of coreceptor usage

For assessment of coreceptor usage, 7 x 10^3^ U87.CD4.CCR5 or U87.CD4.CXCR4 cells were seeded in 96-well plates 24 hours before use and infected, in triplicate, with 5 ng p24gag equivalent of the indicated pseudovirions followed by incubation for 72 h at 37°C. At the end of the incubation period, the cells were harvested, lysed and processed for activity according to the manufacturer’s instructions (Luciferase Assay System; Promega, Madison, WI). Entry, as quantified by relative light unit (RLU), was measured with an MLX microtiter plate luminometer (Dynex Technologies, Inc., Chantilly, VA). For assessment of coreceptor utilization in rhPBMCs, blocking with CCR5 (TAK779) and CXCR4 (AMD3100) inhibitors was performed. Briefly, 5 x 10^6^ SEB-stimulated cells were infected with 200 TCID_50_ of the indicated SHIV in the presence or absence of 1 μM of the chemokine receptor inhibitors. After incubation for 2 hours at 37°C, cells were washed and cultured in 1.5 ml interleukin-2 and appropriate inhibitor-supplemented RPMI medium in each well of a 24-well plate. Culture supernatants were collected over time, and p27^gag^ antigen content was quantified according to the Manufacturer’s instructions (ZeptoMetrix, Buffalo, NY). Percentage blocking at 6 days post-infection was determined by calculating the amount of p27gag antigen production in the presence relative to that in the absence of the inhibitor.

### Detection of antiviral humoral response

The titers of SHIV-specific antibodies were measured by enzyme-linked immunosorbent assay (ELISA) according to the manufacturer’s instructions (GS HIV-1/HIV-2 *PLUS O* EIA; Bio-Rad, Redmond, WA). Endpoint titers were determined as the reciprocal of the highest serum dilution that resulted in an optical density reading greater than the average values obtained with negative human sera plus three standard deviations. Autologous virus neutralization was assessed using TZM-bl cells in 96-well plates. Briefly, equal volumes (50 μl) of the inoculating virus were incubated with 4-fold serial dilutions of heat-inactivated sera from infected macaques for 1 hour at 37°C and then added to cells, in duplicate wells, for an additional 2 hours at 37°C. 100 μl of medium was then added to each well and the virus-antibody (Ab) cultures maintained for 72 hours. Control cultures received virus in the absence of SHIV sera. At the end of the culture period, the cells were lysed and processed for β-galactosidase activity. A neutralization curve was generated by plotting the percentage of neutralization vs serum dilution, and 50% inhibitory dilution (ID_50_) titer was determined using the Prism 4 software (GraphPad, San Diego, CA). Neutralization titers are expressed as the reciprocal of the plasma dilution that inhibited virus infection by 50% (ID_50_). The lowest serum dilution used in the assay was 1:20.

### Immunophenotyping of SHIV-infected cells

Identification of SHIV-infected cells was accomplished with double-label immunohistochemistry performed as previously described with modifications [95,96]. Briefly, lymph node sections were deparaffinized in xylene and rehydrated through graded ethanol to tris-buffered saline (TBS) plus tween 20. Endogenous peroxidase activity was blocked by incubation in 3% H_2_O_2_ in PBS. Antigen retrieval was accomplished by microwave heating sections at 95°C for 20 minutes in citrate buffer (Vector Laboratories, Burlingame, CA), followed by 20 minute cooling, and Dako protein block (Carpinteria, CA) for 10 minutes. The blocked sections were incubated with SIVnef antibody (clone KK75, IgG1; 1:200) overnight at 4°C then reacted with biotinylated secondary antibody (HAM-b, Dako, 1:200) for 30 minutes. Sections were detected using standard avidin-biotin peroxidase complex technique (ABC Elite, Vector Laboratories) and DAB chromagen (Dako). Sections were blocked again for 10 minutes with protein block (Dako) and incubated with Iba-1 antibody (Wako Chemicals, Richmond, VA, rabbit polyclonal, 019–19741, 1:1000) for macrophages for 30 minutes at room temperature followed by biotinylated secondary antibody (GAR-b, Dako, 1:200) for 30 minutes. Sections were detected using standard avidin-biotin alkaline phosphatase complex technique (Vectastain ABC-AP, Vector Laboratories) and Permanent Red (Dako). Slides were counterstained with Mayer’s hematoxylin, rinsed in tap water, coated with Clear Mount (Electron Microscopy Science, Hatfield, PA), air-dried overnight, then coverslipped.

## Competing interests

The authors declare that they have no competing interests.

## Author’s contributions

WR, AG, SW, JB and CCM designed the study. WR, AM, KZ, CH, HK, AG, JB carried out the experiments. WR, SW and CCM analyzed the results and drafted the manuscript. All authors read and approved the final manuscript.
